# Supplementary material for: MetaRibo-Seq measures translation in microbiomes
Source: Nat Commun. 2020 Jun 29;11:3268. doi: 10.1038/s41467-020-17081-z (PMC7324362; doi:10.1038/s41467-020-17081-z)
Supplement: Supplementary file 10 — Supplementary Data 7 [file 41467_2020_17081_MOESM10_ESM.zip › File2/Confidence_High_Taxonomy/362729_out.krona.html]

Javascript must be enabled to view this page.

members
magnitude
magnitudeUnassigned
count
unassigned
taxon
rank

362729\_out

2

2
2
superkingdom

phylum
2
976

200643
2
class

order
2
171549

171552
1
family

genus
1
577309

454154
1

SRS049995\_contig\_number\_17163
species

family
815
1

genus
1
816

817
1

SRS065397\_contig\_number\_69
species
